# Activation of cerebellum and basal ganglia during the observation and execution of manipulative actions

**DOI:** 10.1038/s41598-020-68928-w

**Published:** 2020-07-20

**Authors:** Antonino Errante, Leonardo Fogassi

**Affiliations:** Department of Medicine and Surgery, University of Parma, Via Volturno 39, 43125 Parma, Italy

**Keywords:** Motor control, Basal ganglia, Cerebellum, Motor cortex, Premotor cortex

## Abstract

Studies on action observation mostly described the activation of a network of cortical areas, while less investigation focused specifically on the activation and role of subcortical nodes. In the present fMRI study, we investigated the recruitment of cerebellum and basal ganglia during the execution and observation of object manipulation performed with the right hand. The observation conditions consisted in: (a) observation of manipulative actions; (b) observation of sequences of random finger movements. In the execution conditions, participants had to perform the same actions or movements as in (a) and (b), respectively. The results of conjunction analysis showed significant shared activations during both observation and execution of manipulation in several subcortical structures, including: (1) cerebellar lobules V, VI, crus I, VIIIa and VIIIb (bilaterally); (2) globus pallidus, bilaterally, and left subthalamic nucleus; (3) red nucleus (bilaterally) and left thalamus. These findings support the hypothesis that the action observation/execution network also involves subcortical structures, such as cerebellum and basal ganglia, forming an integrated network. This suggests possible mechanisms, involving these subcortical structures, underlying learning of new motor skills, through action observation and imitation.

## Introduction

It is well known that observation of others’ actions activates, in the brain, the mirror neuron system. Mirror neurons were originally described in the monkey ventral premotor area F5^1–3^ and then in the inferior parietal area PFG^4,5^. These neurons are a distinct class of visuomotor neurons that respond during the observation and the execution of a motor act. Thus, an observed motor act produces in the observer’s brain a motor activation, as if the observer was actually executing it. This mirror neuron matching mechanism^6^ maps the observed action into the observer's own motor representation, allowing a direct understanding of the action goal. Neurophysiological and neuroanatomical studies indicate that the mirror neuron system (MNS) receives its visual input from the superior temporal sulcus (STS)^7–9^. In addition, monkey studies of the last few years provided evidence on the presence of neurons with mirror properties in other areas such as anterior intraparietal (AIP) area^10–12^, dorsal premotor (PMd) cortex^13,14^, ventral prefrontal areas 46 and 12^15^ and mesial premotor area F6^16^.

Up to now it is not known whether subcortical structures contain neurons with properties similar to those of mirror neurons. Anatomical data show that the cerebellum has contralateral connections with multiple cortical areas in both hemispheres, including parietal and premotor areas endowed with mirror properties^17–24^. Furthermore, there are projections from areas of the cortical MNS for hand actions (i.e., area AIP/PFG and PMv) to specific sectors of the putamen, a nucleus of basal ganglia with well-established motor properties^25^. These data suggest the possible involvement of cerebellum and basal ganglia in an extended MNS.

A large number of electrophysiological and brain imaging studies demonstrated the existence of an action observation/execution system also in humans^26–30^. These studies showed that the observation of goal-directed actions done by others results in increased activations not only in visual areas, but also in the inferior parietal lobule (IPL) and in PMv, as well as in the caudal part of the inferior frontal gyrus (IFG)^31,32^. These latter three areas have motor properties and closely correspond to the areas containing mirror neurons in the monkey.

Furthermore, functional MRI (fMRI) studies^30,32,33^ investigating shared cortical activations during both action observation and execution showed common activated voxels not only in the classical parieto-premotor MNS, but also in other cortical areas such as PMd, middle cingulate cortex (MCC), primary somatosensory area (SI), and superior parietal lobule (SPL).

The majority of the above-mentioned studies was focused on cortical circuitry. Only recently, in humans, it has been proposed that aside from the cortical MNS, other subcortical areas, among which the cerebellum and the basal ganglia, might be involved in action observation^30,34–41^. In particular, a very recent study^42^ directly addressed the issue of the localization of cerebellar sectors recruited during grasping action observation, showing activation of specific lobules (VI, VIIb and VIIIa) and suggesting, on the basis of the used task, that human cerebellum is actively involved in perceiving the kinematics of the hand actions of other individuals. Another recent study^43^ comparing observation and execution of a grip force task, showed shared activation in both anterior and posterior cerebellum, with linear and non-linear correlations, respectively, between BOLD signal and the level of applied or detected force. Concerning basal ganglia, interestingly, Ge and colleagues^44^ demonstrated a similar activation of putamen during observation of grasping performed in first- or third-person perspective. They suggest that this visual activation could be combined, in the putamen, with internal simulation of similar actions, in the case of reinforcement learning or imitation.

Many of the above-mentioned studies employed, as observed biological stimuli, grasping actions. Another type of goal-directed hand action consists in manipulative actions. These actions are usually aimed to object exploration, in order to recognise it, in particular in absence of visual feedback, or to use an object for specific tasks, such as, for example, to rotate a handle for opening a door, to move the mouse of the PC, or to unbutton a jacket. In order to execute these types of actions, it is necessary to operate sensorimotor transformations that involve, at the cortical level, specific parieto-frontal circuits. Earlier functional neuroimaging studies, that identified a bilateral cortical network (including dorsal and ventral premotor cortex, SMA, insula, intraparietal cortex) active during haptic object manipulation^45–48^, just reported cerebellar activation. Thus, it is still unclear the specific role of basal ganglia and cerebellum during execution of manipulative actions and nothing is known on the involvement of these structures during observation of manipulation and how they could interact with the main nodes of the MNS.

In the present study, we used fMRI to investigate the specific subcortical activation occurring during observation and execution of manipulative actions in a group of healthy volunteers. The main hypothesis was that, similarly to the cortical structures originally described as mirror areas, specific sectors of the cerebellum and other subcortical structures could show overlapping activation during processing of an observed and executed manipulative action. The results of cortical activation have been reported in a previous paper^49^. In the present work we will focus on the activation of subcortical structures, introducing specific analyses.

## Materials and methods

### Participants

Eighteen human volunteers (10 females; mean age 22.5 years; range 18–25 years) with normal or corrected-to-normal vision, no history of neurological, orthopaedic or rheumatologic disorders, and no drug or alcohol abuse, were recruited. All participants were right-handed according to the Edinburgh Handedness Inventory^50^. Participants had no previous experience with skilled object manipulation, in general, and, in particular, with the specific task used in this study. Individuals with particular manual skills, such as musicians, athletes, typewriters, etc. were excluded from the study. This sample of subjects was the same as that used in our previous study focused on cortical activations^49^. The study was approved by the local ethics committee (Comitato Etico per Parma, University of Parma; code UNIPRMR750v1). All participants gave their informed written consent in accordance with the Declaration of Helsinki. The study was performed in accordance with the guidelines for scientific research of the University of Parma (IT).

### fMRI paradigm and tasks

The fMRI paradigm and procedures for both action observation and execution tasks were previously described in another study focused on cortical MNS activation^49^. Participants laid supine in the bore of the scanner in a dimly lit environment. Visual stimuli were presented by means of a digital goggles system (Resonance Technology, Northridge, CA) (60 Hz refresh rate) with a resolution of 800 horizontal pixels × 600 vertical pixels with horizontal eye field of 30°. Digital transmission of signal to scanner was via optic fiber. Sound-attenuating (30 dB) headphones were used to muffle scanner noise.

### Action observation task

**Figure 1:**
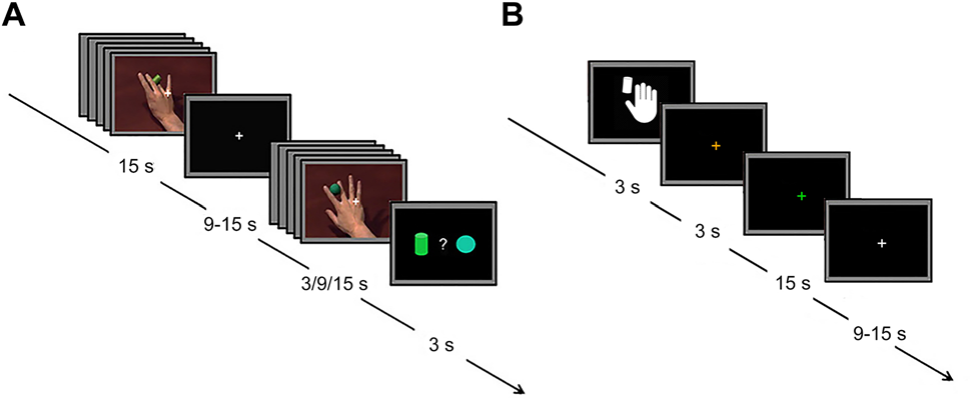
Experimental stimuli and task design. (**A**) Action observation paradigm, presented in two functional runs, made by independent blocks of 15 s, consisting of five randomly presented videos of the same condition. Each block was interleaved with a rest period (9–15 s). In 25% of the blocks, catch-trials were randomly presented after 1, 3 or 5 videos, and participants had to indicate the shape of the previously presented object. (**B**) Action execution paradigm, presented in a single run, alternating execution blocks and baseline periods in a counterbalanced manner among subjects. At the beginning of each block, an instruction cue was presented for 3 s indicating the movement to be performed, then an orange fixation cross appeared in the centre of the black screen, to control for movement preparation. After 3 s, fixation cross colour turned to green instructing subject to perform the movement within a 15 s period.

Experimental stimuli consisted of video clips displaying complex object manipulation performed by an actor with the right hand (Fig. 1A and Suppl. Videos 1–2). Stimuli were recorded using a digital HD Camera and edited using AdobePremiere PRO software (https://www.adobe.com). There were two different conditions: (a) observation of manipulative actions (MAN_OBS); (b) observation of sequences of random finger tapping movements performed with the index, middle, ring or little finger of the right hand (MOV_OBS). This latter condition was used to control for the mere general processing of biological motion. The objects used by the actor for manipulation were a sphere, a cylinder or a coin; specifically, the manipulation sequence consisted in passing the object from the index finger to the ring rotating it. Each action on each object was recorded 5 times, in order to take into account the variability of the performance. For the control condition, 5 random sequences of finger tapping movements performed by the same actor were used. A total of 15 experimental video stimuli (3 manipulated objects × 5 repetitions) and 15 control video stimuli (3 finger movement sequences × 5 repetitions) were presented in the action observation task. All actions and movements were presented to participants in first-person perspective. MAN_OBS and MOV_OBS conditions were presented in two functional runs using a block-design, in counterbalanced manner between subjects. Each run was composed by independent 15 s blocks, each constituted by five videos. Each video had a duration of 3 s. In each run a total number of 8 blocks was presented, 4 blocks per condition. Blocks of stimuli were interleaved by a fixation no-videoclip event (*rest*) lasting 9–15 s, used as baseline, in which participants had to fixate a white cross presented in the middle of a black screen. The fixation cross was maintained during blocks presentation, in order to keep subject’s fixation. Five catch-trials per run were presented, after 1, 3 or 5 videos, and participants had to provide an explicit response, using a response pad positioned on the abdomen. For each catch-trial two objects were presented on the screen, together with a question asking participants to indicate which of the two objects was presented and manipulated by the actor in the last video clip. The catch-trials (3 s each), were followed by a 12 s *rest* period to remove movements-related artefacts. The investigator visually checked subject’s performance, in order to exclude confounding effects due to hand movements during the observation task. Software E-Prime 2 Professional (Psychology Software Tools, Inc.; https://www.pstnet.com) was used both for stimulus presentation and recording of participant response to catch trials.

### Action execution task

In a separate run of the same session, subjects performed an action execution task (Fig. 1B), to define areas involved in hand-object manipulation. Before performing the task, subjects underwent a short training phase inside the scanner, in which they received the instructions and familiarized with a wooden table (dimensions: 40 × 30 cm) to be placed on her/his lap during the MRI acquisition. The table contained a square box containing a small wooden cylinder. During the run, each action started with the subject holding her/his right hand on a starting position near the box and terminated in the same position. There were two conditions: object manipulation (MAN_EXE) and a control condition consisting in the execution of finger tapping movements (MOV_EXE). In the run a total number of 16 blocks were presented, 8 blocks per condition. The task sequence of MAN_EXE was as follows. At the beginning of each block, an instruction cue (a drawing of a hand near a cylinder) was presented for 3 s, then an orange fixation cross appeared in the centre of the black screen, instructing the subject to reach and grasp a cylinder contained in the box. After 3 s, fixation cross colour turned to green instructing subject to perform object manipulation for 15 s. In the control condition, the instruction cue (a drawing of a hand) was presented for 3 s, then the orange fixation cross appeared in the centre of the black screen, instructing the subject to prepare to perform a random sequence of finger tapping movements (frequency 1 Hz). After 3 s, the fixation cross colour turned to green instructing the subject to execute the tapping sequence for 15 s. Both conditions were performed without visual feedback of the own hand. The baseline condition (*rest*) consisted of the static presentation of a white cross in the middle of the screen. The paradigm was administered alternating MAN_EXE blocks, MOV_EXE blocks and rest periods in a counterbalanced manner among subjects.

### Imaging parameters

See Supplementary Information.

### fMRI data preprocessing and analysis

Data preprocessing: see Supplementary Information.

For the normalization of cerebellar cortical data, the T_1_-weighted images were deformed to fit the SUIT template of the human cerebellum using the SUIT toolbox for SPM12^51^ (https://www.diedrichsenlab.org/imaging/suit.htm). The toolbox allows to isolate the cerebellum and creates a mask. For each participant, the mask was manually corrected. Non-linear deformation was then applied to each contrast image. The normalized images were resampled at 2 × 2 × 2 mm^3^ resolution and then smoothed by a 3D convolution with an isotropic Gaussian kernel of 6 mm FWHM.

We report the results of a random effects analysis, in which the corresponding *t*-contrast images (MAN_OBS > Rest, MOV_OBS > Rest, MAN_EXE > Rest, MOV_EXE > Rest) were entered in a flexible ANOVA with sphericity-correction for repeated measures. Within this model, we also assessed the activations resulting from the direct contrasts between the two main conditions and their corresponding control conditions (MAN_OBS > MOV_OBS, MAN_EXE > MOV_EXE, and the reverse contrasts). These contrast analyses were entered in the subsequent conjunction analysis^52^, performed to highlight cortical, cerebellar and other subcortical regions involved in both action observation and execution (MAN_OBS > MOV_OBS AND MAN_EXE > MOV_EXE). Statistical inference was drawn at the cluster level, with a threshold of *P* < 0.001 corrected for multiple comparisons using Family-Wise Error correction (FWE). Local maxima of activations are presented in the stereotaxic space of the MNI coordinate system (see Supplementary Information for details about fMRI data analysis).

In order to obtain the activation maps of dentate nuclei, the whole-brain functional images were masked with a dentate ROI^53^ (see Fig. 6A) using the explicit mask procedure in the second level analysis.

### Cortical, cerebellar and further subcortical ROI analyses

To investigate possible differences between BOLD activations in selected cortical, cerebellar and further subcortical areas, we performed three different Region of Interest (ROI) analyses.

ROIs were defined according to the following criteria: (1) significant activations following the conjunction between observation and execution of manipulation (MAN_OBS > MOV_OBS AND MAN_EXE > MOV_EXE); (2) shared activations from conjunction, surviving a threshold of *P* < 0.001, FWE corrected at cluster level, cluster dimensions > 42 voxels. To avoid any circularity issue in ROI selection^54^, we did not rely directly on the functional data but defined ROI at single-subject level. The subject-specific location of each ROI was guided both anatomically and functionally: anatomically, using the probabilistic cytoarchitectonic maps implemented in the SPM anatomy toolbox^55^, functionally, based on activations coordinates reported in previous metanalyses of fMRI studies on action observation and execution^31,32^. At cortical level, our analysis included 6 areas in the left hemisphere, known to be involved in the processing of action observation and execution, belonging to the extended MNS: (a) Left IPL (x =  − 58, y = − 44, z =  + 40) defined according to the anatomical studies of Caspers and colleagues^56,57^ and the metanalysis of Caspers and colleagues^31^; (b) Left SPL (x = − 20, y = − 67, z =  + 63) labeled as Area 7A in the SPM Anatomy toolbox according to the study of Scheperjans and colleagues^58^; (c) Left IPS (x = − 32, y = − 59 , z =  + 51), labeled as Areas hIP2/hIP3 according to anatomical studies of Choi and colleagues^59^ and Scheperjans and colleagues^58^; (d) Left Area 44 (x = − 53 , y =  + 7, z =  + 22) according to Amunts and colleagues^60,61^, that also includes PMv cortex; (e) Left PMd (x = − 26, y = − 8, z =  + 60) defined according to the anatomical study of Geyer^62^, including not only the PMd cortex, laterally, but also part of the SMA and the pre-SMA, medially; (f) Left SI (x = − 45, y = − 34, z =  + 60) according to the study of Geyer and colleagues^63^.

In order to better explore the contribution of subcortical structures, the analyses on cerebellar and the other subcortical ROIs were carried out on both hemispheres. Concerning cerebellum, 6 ROIs were defined in both hemispheres on the basis of probabilistic cerebellar atlas^51^, namely: (a) Left Lobule VI (x = − 22, y = − 63, z = − 19); (b) Left Lobule VIIIa (x = − 28, y = − 60, z = − 48); (c) Left Lobule VIIIb (x = − 19, y = − 54, z = − 50); (d) Right Lobule VI (x =  + 24, y = − 61, z = − 21); (e) Right Lobule VIIIa (x =  + 27, y = − 62, z = − 50); (f) Right Lobule VIIIb (x =  + 19, y = − 55, z = − 51).

Concerning the other subcortical structures, 4 different ROIs were defined in the globus pallidus (GP) and red nucleus (RN) as follows: (a) Left GP (x = − 18, y = 0, z =  + 1); (b) Right GP (x =  + 17, y = − 4, z =  + 4); (c) Left STN/RN (x = − 8, y = − 17, z = − 8); (d) Right STN/RN (x =  + 8, y = − 18, z = − 8), according to the 7 T MRI probability maps provided by the Atlas of Basal Ganglia (ATAG)^64,65^, https://www.nitrc.org/projects/atag/.

For each subject we defined a sphere using MarsBaR software for SPM (https://marsbar.sourceforge.net/), with maximum cluster size of 2 mm radius around the peak activation, within each anatomically defined region. Then, for each subject we extracted separately in each ROI the average BOLD signal change across all significant voxels using the SPM Rex Toolbox (https://web.mit.edu/swg/rex). All subjects showed significant activations in the ROIs considered for the analyses.

In the cortical ROI analysis, the PSC within each ROI was compared between the action observation and action execution conditions, using a 6 × 2 analysis of variance (ANOVA) with *ROI* and *Condition* as repeated measures factors. A similar approach was employed in the cerebellar ROI analysis, using a 6 × 2 ANOVA with *ROI* and *Condition* as repeated measures factors. For subcortical ROI analysis, a 4 × 2 ANOVA with *ROI* and *Condition* as repeated measures factors was calculated. To investigate significant differences, post-hoc comparisons were computed by using paired-sample *t*-tests with Bonferroni correction for multiple comparisons.

## Results

**Figure 2:**
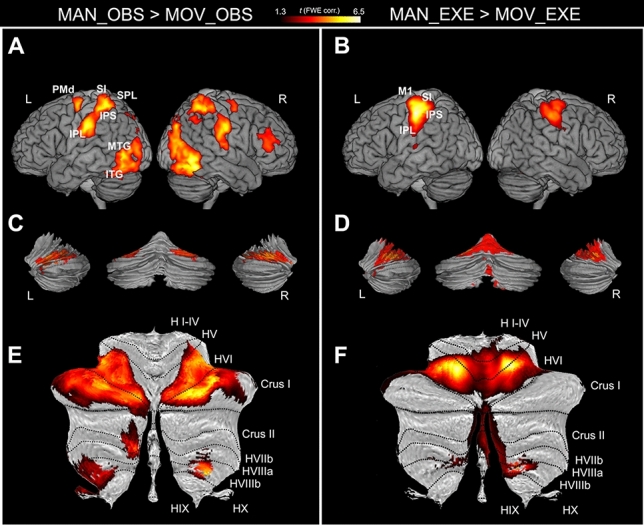
Cortical and cerebellar activations related to the contrasts between experimental and control conditions (MAN_OBS > MOV_OBS and MAN_EXE > MOV_EXE). (**A**,**B**) 3D MNI brain template ch2^126^ (MRIcron software; https://people.cas.sc.edu/rorden/mricron/index.html) left view, right view; (**C**,**D**) 3D cerebellar template (Caret, Computerized Anatomical Reconstruction and Editing Tool Kit 5.61^127,128^ https://brainvis.wustl.edu/wiki/index.php/Caret:About); left view, posterior view and right view); (**E**,**F**) flat map of cerebellum (SUIT, spatially unbiased atlas template of the cerebellum^51^, https://www.diedrichsenlab.org/imaging/suit.htm). All activations are rendered with a threshold of *P* < 0.001 (FWE corrected at cluster level). L, left hemisphere; R, right hemisphere.

**Figure 3:**
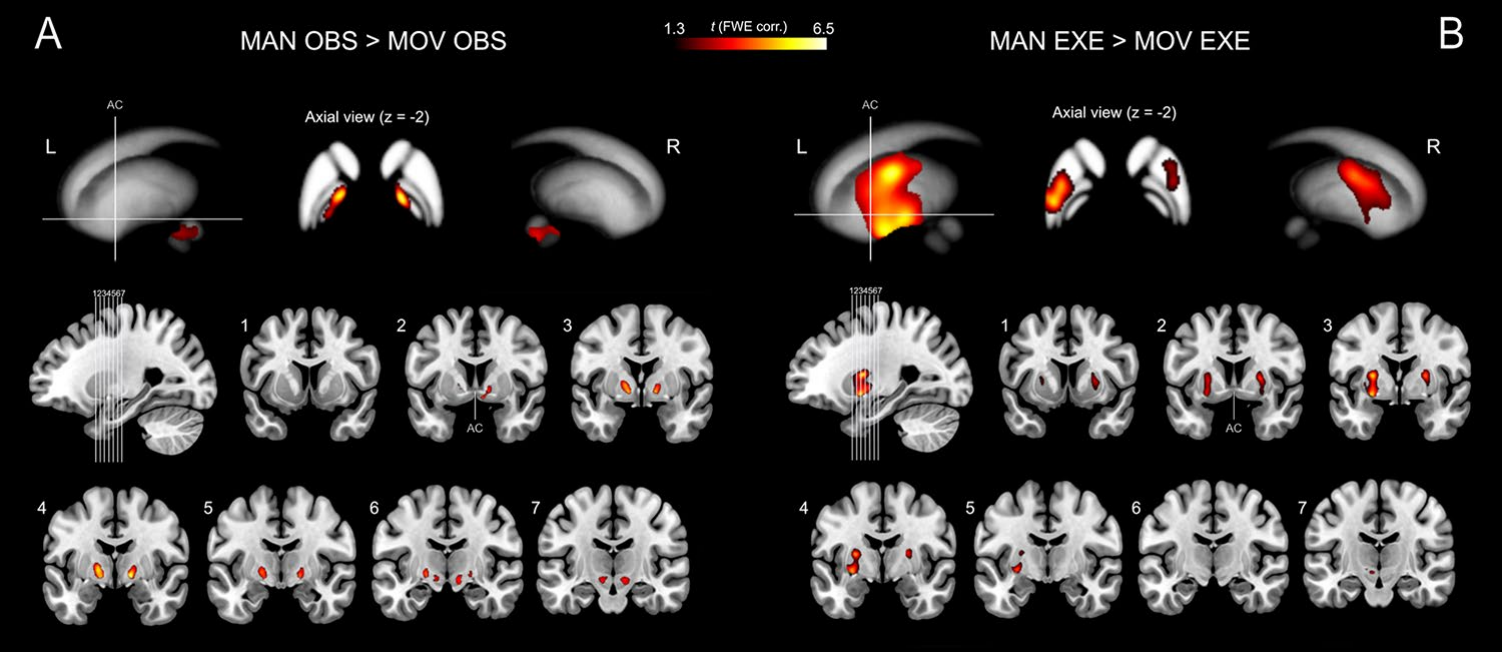
Activations of basal ganglia and other subcortical structures related to the contrasts between experimental and control conditions (**A**) MAN_OBS > MOV_OBS and (**B**) MAN_EXE > MOV_EXE. Activation are shown on 3D basal ganglia template (ATAG, Atlas of the basal ganglia^65^; https://www.nitrc.org/projects/atag/; left view, right view and axial view) and seven parasagittal representative sections from ch2 template^126^ (MRIcron software; https://people.cas.sc.edu/rorden/mricron/index.html). AC = anterior commissure.

In order to have a more complete description of the network activated during the execution and observation conditions, the activations of both cortical (Fig. 2A,B) and subcortical (Figs. 2C–F and 3A,B) structures are presented. Statistical details and MNI coordinates of activation peaks are reported in Table 1.

**Table 1:** Statistical values for GLM group analysis related to the action observation and execution tasks.

Contrast	Brain structure	Side	*k*	Z	Local maxima (MNI)		
					x	y	z
MAN_OBS > MOV_OBS	Inferior parietal lobule	L	51,586	7.10	− 50	− 22	+ 32
		R		6.12	+ 62	− 22	+ 30
	Superior parietal lobule	L		6.16	− 32	− 46	+ 64
		R		5.51	+ 34	− 52	+ 60
	Intraparietal sulcus	L		3.70	− 34	− 54	+ 56
		R		4.38	+ 40	− 44	+ 50
	Postcentral gyrus	L		6.07	− 38	− 38	+ 56
		R		6.14	+ 32	− 36	+ 52
	Fusiform gyrus	L		6.25	− 30	− 52	− 14
		R		6.47	+ 34	− 48	− 14
	Cerebellum (Lobule VI)	L		5.96	− 28	− 54	− 18
		R		6.73	+ 28	− 54	− 20
	Cerebellum (Lobule VIIIb)	L	179	4.47	− 16	− 54	− 52
		R	124	5.37	+ 16	− 52	− 52
	IFG (pars Triangularis)	R	1599	4.63	+ 40	+ 32	+ 16
	IFG (pars Opercularis)	R		4.51	+ 58	+ 14	+ 32
	Middle frontal gyrus	R		4.49	+ 44	+ 46	+ 16
	Precentral gyrus	L	1,309	6.51	− 30	− 12	+ 64
		R	938	6.14	+ 28	− 10	+ 58
	Thalamus	L	472	4.26	− 18	− 27	+ 1
		R	2,384	4.88	+ 26	− 26	0
	Globus Pallidus	L		4.22	− 15	− 2	+ 2
		R		4.78	+ 13	− 3	− 6
	Insula	L	95	6.36	− 38	− 6	+ 14
		R	491	6.33	+ 38	+ 0	+ 11
	Cingulate cortex	L	2,457	5.15	− 6	− 30	+ 24
		R		6.06	+ 6	− 22	+ 26
MAN_EXE > MOV_EXE	Cerebellum (Lobule VI)	L	18,759	> 7.7	− 20	− 58	− 20
		R		> 7.7	+ 20	− 52	− 24
	Cerebellum (Lobule VIIIb)	L	56	3.25	− 20	− 54	− 50
		R	68	3.25	+ 18	− 62	− 50
	Postcentral gyrus	L	7,715	> 7.7	− 50	− 20	+ 54
		R	5,073	6.86	+ 50	− 24	+ 50
	Precentral gyrus	R		6.69	+ 38	− 18	+ 56
	Thalamus	L	117	4.61	− 16	− 22	− 2
	Posterior medial frontal cortex	R	602	4.19	+ 2	− 6	+ 50
	Middle Cingulate cortex	R		4.08	+ 6	+ 2	+ 36
	Insula	L	481	4.06	− 38	− 16	+ 8
		R	45	4.02	+ 38	0	+ 12
	SII	L		3.69	− 54	− 20	+ 12
	Putamen	L	85	4.20	− 28	− 6	− 6
		R	52	3.58	+ 24	− 2	+ 8

Local maxima corresponding to the activation maps shown in Figs. 2 and 3 are given in MNI standard brain coordinates. Significant threshold is set at *P* < 0.001, FWE-corrected for multiple comparisons at cluster-level. Most probable anatomical regions are derived from Anatomy Toolbox 1.7^55^ and listed in “Brain structure” column. *k* = main cluster size.

### Brain activations during observation of object manipulation

Activations of cerebellar cortex during the contrast MAN_OBS > MOV_OBS are shown in Fig. 2C,E. The activation pattern was largely symmetrical in both cerebellar hemispheres, with clusters of activity in lobules V, VI, crus I, VIIIa and VIIIb, although some of them seem more lateralized to the right hemisphere, ipsilateral to the observed hand, such as, for example, the local maxima in lobule VI.

Other subcortical regions showing consistent bilateral activation were basal ganglia (Fig. 3A), including internal and external segments of the globus pallidus (GP) and the subthalamic nucleus (STN), the thalamus, very likely corresponding to the pulvinar and the ventral posterolateral (VPL) nuclei, and the red nucleus (RN).

### Brain activations during execution of object manipulation

The cerebellar activation during the execution of manipulative actions versus simple finger tapping movements (Fig. 2D,F) included a large bilateral cluster in lobules IV, V, VI, presumably corresponding to the anterior sensorimotor representation of the hand^66,67^, and other clusters located in lobules VIIIa and VIIIb, very likely corresponding to the secondary motor representation^66,67^.

Further subcortical activations were found in the basal ganglia (Fig. 3B), including the putamen, the external segment of GP and the STN bilaterally, the thalamus, very likely corresponding to the left pulvinar oralis, and the RN bilaterally^64,68^.

### Conjunction analysis between observation and execution

**Figure 4:**
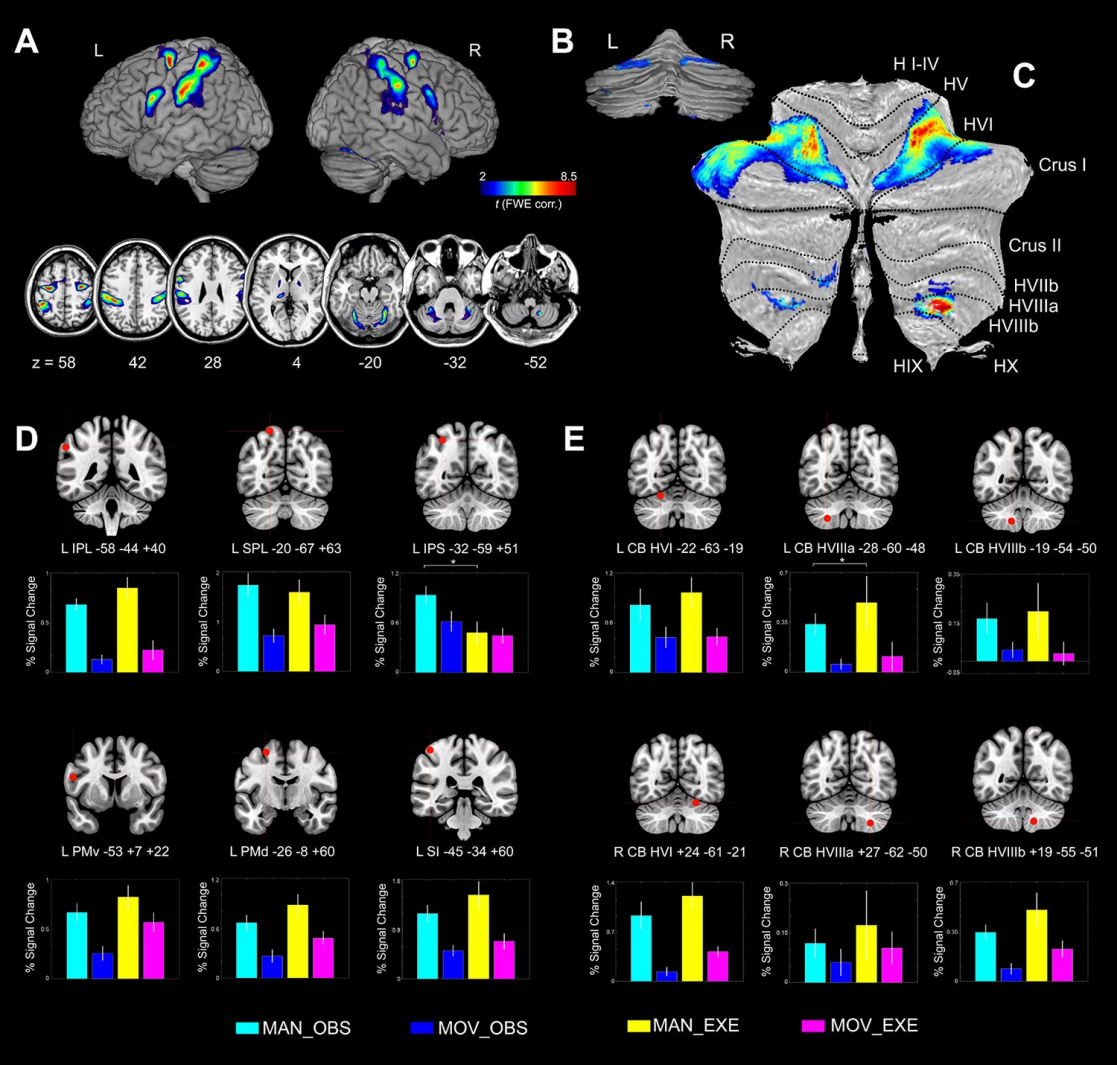
(**A**–**C**) Statistical parametric map showing the results of the conjunction analysis between (MAN_OBS > MOV_OBS) AND (MAN_EXE > MOV_EXE) contrasts (cerebral cortex and cerebellum). Shared activation voxels are rendered into (**A**) 3D MNI brain template and 7 representative axial slices from ch2 template^126^ (MRIcron software; https://people.cas.sc.edu/rorden/mricron/index.html), (**B**) 3D posterior view of the cerebellum (Caret, Computerized Anatomical Reconstruction and Editing Tool Kit 5.61^127,128^, https://brainvis.wustl.edu/wiki/index.php/Caret:About), and (**C**) flat map of the cerebellum (SUIT, spatially sunbiased atlas template of the cerebellum^51^, https://www.diedrichsenlab.org/imaging/suit.htm). (**D**, **E**) Results of the cortical and cerebellar ROIs analyses. The histograms show the averaged magnitude of activation (% signal change) in each ROI. Above each histogram the corresponding ROI is presented as red coloured sphere. Vertical lines in the histograms indicate standard error mean.

In order to investigate significant voxels that presented shared activation during both MAN_OBS > MOV_OBS AND MAN_EXE > MOV_EXE, we used a conjunction analysis^52^. At cortical level, shared voxels were found in the main nodes of the parieto-frontal MNS (Fig. 4A). Statistical details and MNI coordinates of significant clusters revealed by conjunction analysis are reported in Table 2. Figure 4B shows that, relative to control conditions, shared voxels with increased activity during observation and execution of manipulative actions were present in lobules V, VI, crus I, VIIIa and VIIIb, bilaterally. Figure 4C shows a flat map of significant activations, computed using SUIT^51^ toolbox for SPM12 (*P* < 0.001 FWE corrected at cluster level; *t* > 2). The anterior cluster included lobule V, VI and the crus I, with local maxima located in the hemispheric zone of lobule VI. Since the cluster presented multiple activation peaks, we performed the subsequent ROI analysis focusing on lobule VI (see below).

**Table 2:** Statistical values for the conjunction analysis between observation and execution conditions.

Contrast	Brain structure	Side	*k*	Z	Local maxima (MNI)		
					x	y	z
Conjunction	Inferior parietal lobule	L	7,199	7.18	− 40	− 36	+ 50
		R	5,639	5.74	+ 37	− 36	+ 50
	Supramarginal gyrus	L		6.98	− 50	− 22	+ 36
		R		6.10	+ 50	− 20	+ 36
	Postcentral gyrus	L		6.26	− 56	− 20	+ 38
		R		7.13	+ 56	− 18	+ 40
	Superior parietal lobule	L		3.41	− 18	− 52	+ 68
		R		4.10	+ 18	− 54	+ 68
	Cerebellum (Lobule VI)	L	1,209	5.60	− 26	− 68	− 22
		R	597	5.68	+ 24	− 70	− 20
	Cerebellum (Lobule VIIIb)	L	42	3.91	− 14	− 56	− 50
		R	91	4.66	+ 18	− 54	− 52
	Cerebellum (Lobule VIIIa)	L		4.10	− 12	− 68	− 46
		R		3.92	+ 16	− 64	− 50
	Precentral gyrus	L	1907	6.33	− 32	− 8	+ 60
		R	1547	5.89	+ 28	− 8	+ 54
	IFG (BA44)	L	694	5.88	− 58	+ 6	+ 28
	IFG (pars opercularis)	R	435	4.83	+ 58	+ 12	+ 28
	Thalamus	L	299	4.91	− 18	− 26	+ 2
	Globus pallidus	L	82	3.64	− 18	− 2	+ 4
		R	59	3.06	+ 18	− 4	+ 4

**Figure 5:**
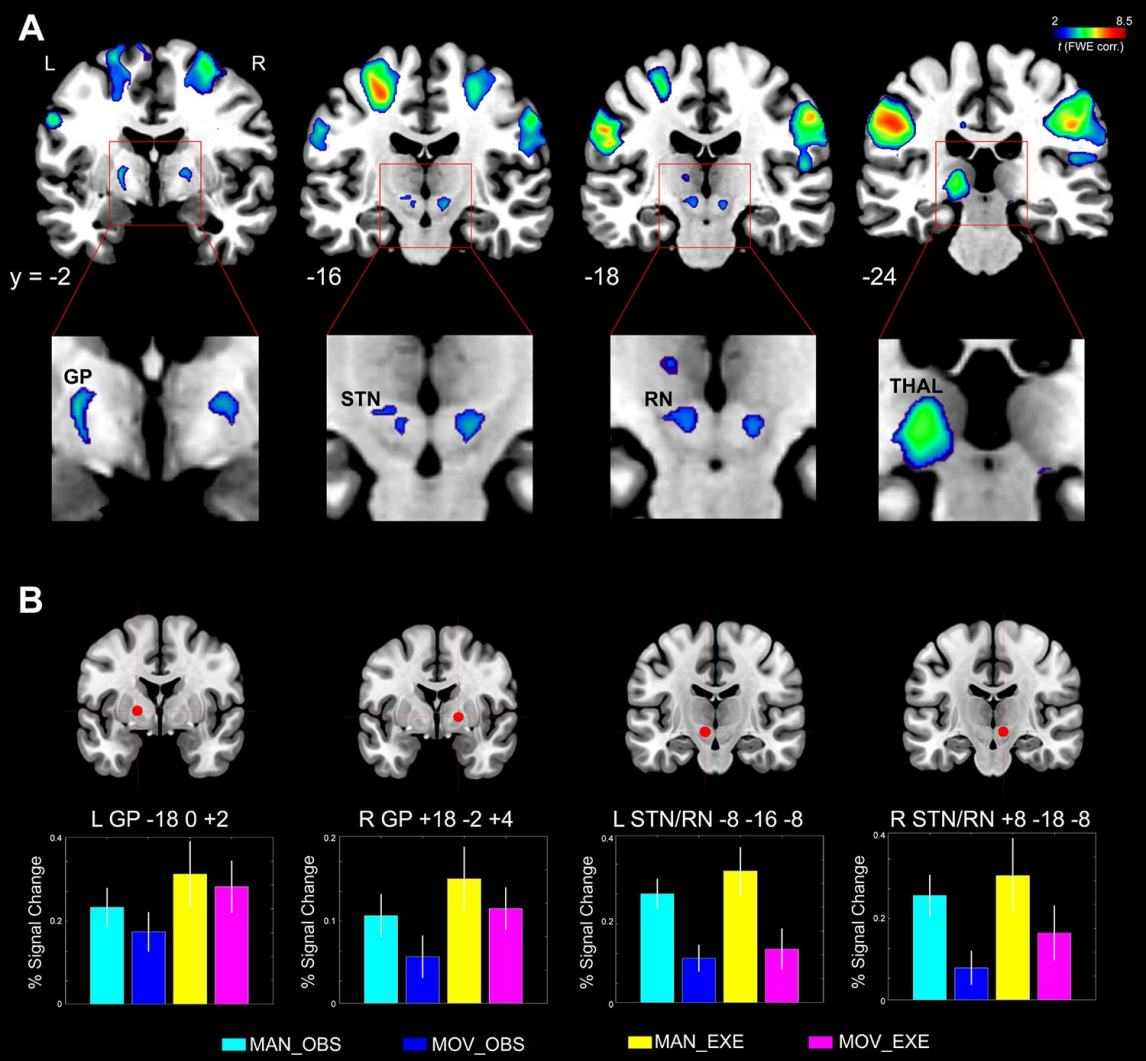
(**A**) Statistical parametric map showing the results of the conjunction analysis between (MAN_OBS > MOV_OBS) AND (MAN_EXE > MOV_EXE) contrasts (subcortical structures). Shared activation voxels are rendered into 4 representative coronal slices (MRIcron software; https://people.cas.sc.edu/rorden/mricron/index.html). (**B**) Results of the ROI analysis in subcortical structures. The histograms show the averaged magnitude of activation (% signal change) in each ROI. Above each histogram the corresponding ROI is presented as red coloured sphere. Vertical lines in the histograms indicate standard error mean.

Local maxima corresponding to the activation maps shown in Figs. 4 and 5 are given in MNI standard brain coordinates. Significant threshold is set at *P* < 0.001, FWE-corrected at cluster-level. Most probable anatomical regions are derived from Anatomy Toolbox 1.7^55^ and listed in “Brain structure” column. *k* = main cluster size.

Shared voxels for observation and execution have also been found in subcortical areas belonging to the left and right GP and STN, left thalamus, very likely corresponding to the pulvinar, and RN bilaterally (Fig. 5A).

### Dentate nucleus activation

**Figure 6:**
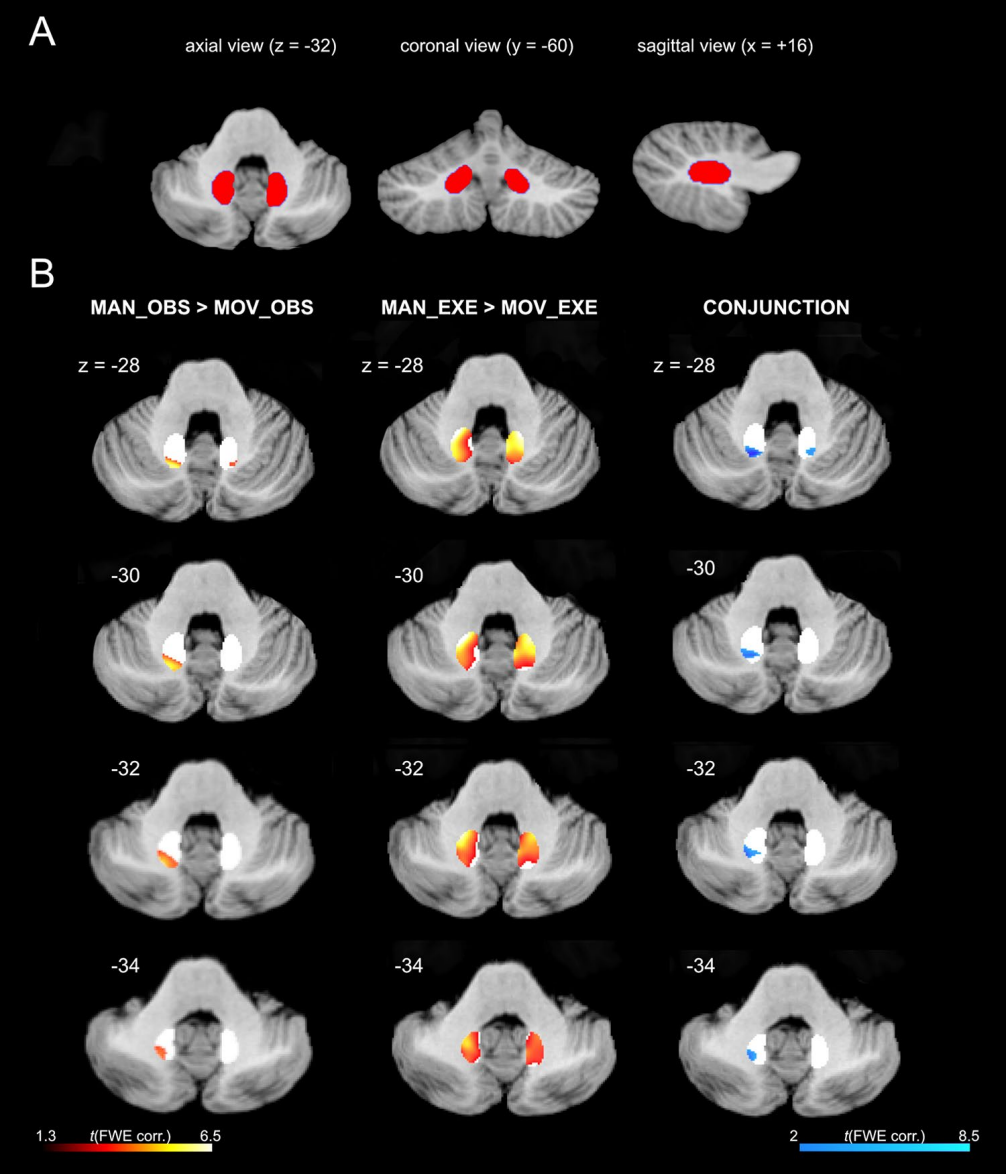
Dentate activations. (**A**) Dentate ROI mask^53^ used to investigate dentate nuclei activations. (**B**) Statistical parametric maps showing the dentate nuclei activations during the contrasts MAN_OBS > MOV_OBS, MAN_EXE > MOV_EXE and the conjunction analysis between observation AND execution conditions. Activation corresponding to each contrast is overlaid in four representative axial slices^51^ (from z = − 28 to − 34).

The dentate ROI mask used to investigate the activations of these deep cerebellar nuclei are shown in Fig. 6A. Dentate activations are shown superimposed on the MNI cerebellar template^51^ (axial view) in Fig. 6B. Suprathreshold clusters of activation were present both in observation and execution conditions contrasted with their corresponding controls. In the MAN_OBS > MOV_OBS contrast, symmetric clusters were detected bilaterally, although highest *t*-values and larger cluster size were present on the left. In the MAN_EXE > MOV_EXE contrast the activation of dentate was more symmetrical as compared to action observation condition. To further investigate the presence of shared voxels between observation and execution condition in these deep cerebellar nuclei, we mapped the data obtained using the conjunction analysis. This analysis shows the presence of shared voxels with a larger cluster in the left hemisphere with respect to the right one (Fig. 6B).

### ROI analyses results

The most important comparison was that relative to observation (MAN_OBS) versus execution (MAN_EXE) of manipulative actions, investigated also within specific ROIs localized using anatomical reference and previous cytoarchitectonic studies (see “Cortical, cerebellar and further subcortical ROI analyses”). The averaged percent signal change (PSC) within cortical ROIs (histograms of Fig. 4D) have been analysed at group level using a 6 × 2 analysis of variance (ANOVA) with *ROI* and *Condition* as repeated measures factors. Post-hoc comparisons were computed by using paired-sample *t*-tests with Bonferroni correction for multiple comparisons (alpha set to *P* < 0.05 corr.). The ROI analysis revealed a significant main effect for ROI [*F* (5, 80) = 9.38, *P* < 0.0001, *η*^2^ = 0.36], with higher BOLD response within the SPL compared to IPL (*P* < 0.001), IPS (*P* < 0.001), PMd (*P* < 0.001) and PMv (*P* < 0.001), but not when compared to SI. Concerning the factor *Condition,* we did not find any significant effect in cortical ROIs, indicating similar PSC during both observation and execution. In addition, the interaction between *ROI* × *Condition* was significant [*F* (5, 80) = 5.57, *P* < 0.001, *η*^2^ = 0.25]. Post-hoc comparisons showed that BOLD level differed only in IPS ROI, with increased activation during observation than execution (*P* < 0.004).

Concerning the assessment of BOLD change in the cerebellar ROIs (see histograms of Fig. 4E), the 6 × 2 ANOVA with *ROI* and *Condition* as repeated measures factors revealed a significant effect for *ROI* [*F* (5, 80) = 18.60, *P* < 0.0001, *η*^2^ = 0.53]. However, no effect was found for *Condition*, similarly to the cortical ROI analysis, indicating no statistical difference between observation and execution activations. Post-hoc comparisons (Bonferroni) indicated that left lobule VI was more strongly activated than left lobule VIIIa (*P* < 0.001), VIIIb (*P* < 0.001), and right lobule VIIIa (*P* < 0.001). In addition, also the activation within right lobule VI was higher than that in left lobules VIIIa (*P* < 0.001), VIIIb (*P* < 0.001), and right lobules VIIIa (*P* < 0.001), VIIIb (*P* < 0.001). The interaction *ROI* × *Condition* was also significant [*F* (5, 80) = 4.57, *P* < 0.001, *η*^2^ = 0.22]. Post-hoc comparisons showed similar BOLD activity for observation and execution of manipulative actions in cerebellar ROIs, except for the left lobule VIIIa, in which increased activation was present during execution compared to observation (*P* < 0.004).

At a further subcortical level (Fig. 5B), the 4 × 2 ANOVA with *ROI* and *Condition* as repeated measures factors indicated a specific main effect for *ROI* [*F* (3, 51) = 3.34, *P* < 0.02, *η*^2^ = 0.16] but not for *Condition* (*P* = 0.38; n.s.), indicating a general difference in BOLD change between ROIs but not between observation and execution conditions. Also the interaction *ROI* × *Condition* was not significant.

## Discussion

In the present study we analysed, using fMRI, brain activations of a group of healthy volunteers during the observation and execution of complex manipulative actions. The results, mainly focused on subcortical structures, indicate that: (a) both observation and execution of manipulative actions versus simple finger tapping movements activate subcortical structures, including basal ganglia, thalamus and cerebellum; (b) in both conditions, the activations are mainly bilateral, although in the cerebellum activation intensity is higher in the right hemisphere; (c) shared voxels between observation and execution of manipulation showing increased activity with respect to control conditions are present in cerebellar lobules V, VI, crus I, VIIIa and VIIIb, in GP and STN of basal ganglia, in the thalamus and in the RN; (d) ROI analyses performed for comparing the two main conditions confirmed the general results of the conjunction analysis, revealing a significant differential activation only in the IPS and lobule VIIIa of the cerebellum.

### Activation of the cortical mirror neuron system

Cortical activation during the observation of manipulative actions versus simple finger tapping movements has been previously reported in a separate manuscript^49^. Thus, we will limit to discuss here the comparison between the cortical activation during action observation with that during action execution obtained using the new conjunction and ROI analyses included in the present work. The conjunction analysis reveals an activation of both dorso-dorsal circuit, connecting SPL with PMd, and dorso-ventral circuit, connecting IPL with PMv. These two circuits have been originally described as separate modules for reaching and grasping in both monkeys and humans^33,69,70^. More recent studies in humans reveal an activation of the dorsal circuit also for grasping actions^71,72^. Furthermore, the use of paradigms that allow to study independently specific aspects of actions, such as the action goal and some aspects of hand kinematics, suggest that, on the one side, both circuits are involved in decoding hand actions, on the other the dorso-dorsal circuit appears to have a more relevant role in decoding hand action kinematics^49,73–76^. The coding of the kinematic features is likely related to an automatic motor resonance of the parietal and premotor regions involved in this type of elaboration. In principle, this activation could be even higher when subjects are instructed to imitate observed actions^29,77,78^. The ROI analysis also corroborates the conjunction analysis data.

### Activation of the cerebellum and its role within the mirror neuron system

Based on clinical observations and on neuroanatomical and electrophysiological studies, cerebellum has been traditionally considered as a brain structure crucially involved in motor control and motor learning^19,79–83^. This role is confirmed also by neuroimaging studies with healthy subjects performing motor tasks^84,85^. On the other hand, recent works indicate that the cerebellum is engaged not only in motor control, but also in cognitive, perceptual and social functions^84,86–89^. These studies show differential activations when sensorimotor or cognitive tasks are employed, respectively: the former mostly activate the anterior lobules, with a secondary representation within lobule VIIIa and VIIIb; linguistic tasks mostly activate the right posterolateral cerebellum, with a more anterior activation when articulation is involved; working memory tasks show activations that overlap with those elicited by language; executive functions activate different cerebellar regions depending on the nature of the task. Motor tasks reveal also a somatotopic organization in the cerebellar cortex, with the different effectors represented in partially segregated sectors of lobules V, VI and VIII^89,90^.

A few studies reported the activation of cerebellum during action observation, but mostly as an additional finding not addressed as a main focus of the work^30,34–36,49,91^. However, a very recent work^42^ in which healthy subjects were required to observe reaching-grasping actions clearly shows activation of cerebellar lobules VI, VIIb and VIIIa in both hemispheres. In order to investigate the existence of a mirror matching mechanism^6^ in the cerebellum, it is necessary to demonstrate the presence of shared activation between action observation and execution. A previous work^30^ showed, in the cerebellum, bilateral shared voxels for observation and execution of hand, mouth and foot actions, with a higher percentage of hand-related voxels, although the precise localization was not specified. The results of our study on observation/execution of manipulative actions generally confirm the activation reported in this literature. In addition, conjunction analysis specifically localizes the lobules showing shared activations, corresponding to cerebellar sectors compatible with those classically considered as part of the motor loop. A shared activation is also observed in the dentate nucleus, that constitutes the main output channel of the cerebellum to the motor thalamus and the motor cortex^92,93^.

Dentate nucleus activation has been previously reported in humans during sequential movements of fingers and tongue^94^, force control^95^ and oculo-manual coordination control^96^. The function of this structure appears to be correlated with voluntary coordination or movement correction, even in the case in which sensory feedback is absent^97^.

Although most of activation is shared between the two conditions, the conjunction analysis reveals also that some sectors of cerebellum are active only during action observation condition, such as crus I—a region activated by language and working memory tasks^84^—or only during action execution condition, such as the most medial part of the vermis of lobules IV, V, VI and VIII. The first finding suggests that the used task can recruit also high order cortico-cerebellar circuits, such as those involving prefrontal cortex; the second one indicates that some cerebellar motor sectors are exclusively devoted to actual motor control.

Altogether, these data indicate that a mirror matching mechanism is present also in the cerebellar circuitry. What is the possible contribution given by cerebellum to the mirror circuit during action observation? It has been proposed that the cerebellum, during movement execution, can be involved in both forward and inverse models^40,41,98–101^ thanks to its reciprocal, indirect connections with several areas of cerebral cortex, such as primary motor, premotor and posterior parietal cortices^88,102–106^. The activation seen during observation seems to replicate this kind of processing, although in this case participants do not perform any voluntary movement. This would suggest that the motor system, in particular when the observed action is quite complex, performs an internal simulation of the action that also involves subcortical structures. The activation of the dentate nucleus and the thalamus suggests that this simulation forms a closed loop, in which, probably, also the prediction of sensory consequences is included. This loop can generate, as in actual execution, an update of the cortical hand motor representations, based on the consequences of the observed action. The role of cerebellum in this kind of network is particularly relevant because it is related to the processing of sequential and temporal dynamics of the specific manipulation^40,107,108^.

An interesting parallel of our findings is represented by studies on motor imagery, because also in this latter condition there is no overt execution, but the motor system is activated, including the lobules VI and VII of cerebellum^109^. In line with this, patients with cerebellar lesions have deficits in performing motor imagery and in creating action sequences starting from observed isolated pictures showing the motor acts composing these sequences^110^.

A direct consequence of the cerebellar engagement during action observation can be its possible role of mirroring specific aspects of the observed actions during observation-based motor learning, in which the movement pattern of the observed agent can constitute a predictive model for the observer to match her/his movement execution pattern.

### Activation of basal ganglia and their role in the mirror neuron system

The basal ganglia, as cerebellum, have been considered as brain structures crucially involved in motor control and motor learning, as well as in some cognitive functions^21,23,111,112^. While the literature in humans clearly shows basal ganglia activation during movement execution^111^, very little is known on their activation during action observation. Only one electrophysiological work^39^ focuses on the STN, showing that this nucleus presents an EEG beta-reduction and a coherence with cerebral cortex during both movement observation and execution, this effect being stronger during the latter condition.

The results of our study confirm that during action execution there is an activation of basal ganglia, including the classical motor sector of the putamen^111,113,114^, plus GP and STN. During passive observation there is a similar activation of these two latter nuclei. The conjunction analysis confirms the presence of shared voxels in most of the regions of GP and STN activated by both tasks. Interestingly, the motor sector of the putamen is not activated by the observation of hand manipulation. Since apparently the first station of the basal ganglia activated by observation is GP, it is possible that action observation condition enhances the activity of hyper-direct pathway (connecting motor cortex with the STN)^39,40^ rather than the other (direct and indirect) pathways, usually involved during actual movement. This would likely bring, as final outcome, to an inhibition of the thalamic motor nuclei, that is plausible since during observation overt movement must be blocked^115^. On the contrary, when the movement is overtly executed, the putaminal activation induces an activation of the direct pathway, whose outcome is the facilitation of specific types of movements. The activation by action observation of the STN confirms also the finding of Alegre and colleagues^39^, who showed local field potentials changes in STN during observation of wrist movements.

Another possible interpretation of the absence of putaminal activation during action observation is that this activation depends on the type of task. In line with this idea, Ge and colleagues^44^ suggested an involvement of this structure during the observation of grasping. In this latter case the activation of the putamen could be explained by the presence of grasping action in the motor repertoire, while the type of manipulation task used in the present study was unfamiliar to participants, thus less represented in the putamen.

The task used in this experiment requires, in the agent, several processes, including sensorimotor integration occurring when the object is moved between the different fingers, planning of a specific motor sequence and focused attention for avoiding object falling. In a hypothetical learning context, at the beginning the individual makes this task step-by-step, while at the end the sequence consists in a fluid concatenation of finger flexions and extensions. During observation without instruction to imitate, very likely the most important function for basal ganglia is to inhibit automatic movement execution. In the case of observational learning, the observation phase should produce an activation more similar to that found during execution^116^. The role of this activation might be related to the automatic selection and facilitation of motor acts at the level of the cortical parieto-premotor mirror circuit. This mechanism would facilitate motor learning and the building of new motor patterns.

## Conclusions and possible clinical implications

The results of the present investigation reveal that during observation of complex manipulation both basal ganglia and cerebellum are activated. It is noteworthy that recently the concept of independence of the cortico-basal ganglia and cortico-cerebellar loops has been revised on the basis of neuroanatomical and functional data revealing a reciprocal influence between these two subcortical structures^23,117–119^. This new perspective opens new lines of interpretation of normal function in healthy individuals and of altered function in patients with neurological diseases due to lesions or degenerations of these structures, including functions related to action observation, such as imitation and motor learning. For example, in patients with Parkinson’s disease (PD) it has been shown that the Action Observation Therapy (AOT)^120–122^, based on observation of actions followed by their immediate reproduction, produces an improvement of motor symptoms^123–125^. Data on deep brain stimulation of STN of PD patients demonstrate that there is a reduced cerebellar cortex hyperactivity and improvement of motor function, likely related to a facilitation of deep cerebellar nuclei. It is possible that the effect of the AOT are not simply related to changes in basal ganglia and their cortical targets, but also to modifications in the cerebellar-thalamo-basal ganglia-cortical loop.

## Supplementary information

Supplementary Information 1.

Supplementary Video 1.

Supplementary Video 2.

## Data Availability

All data generated or analysed during this study are included in this published article (and its Supplementary Information files).
