# Supplementary material for: Activation of cerebellum and basal ganglia during the observation and execution of manipulative actions
Source: Sci Rep. 2020 Jul 20;10:12008. doi: 10.1038/s41598-020-68928-w (PMC7371896; doi:10.1038/s41598-020-68928-w)
Supplement: Supplementary file 1 — Supplementary Information 1. [file 41598_2020_68928_MOESM1_ESM.docx]

Activation of cerebellum and basal ganglia during the observation and execution of manipulative actions

Running Title

Cerebellum and Basal Ganglia in action observation and execution

Antonino Errante^1^ and Leonardo Fogassi^1*^

*^1^ Department of Medicine and Surgery, University of Parma, Parma, 43125, Italy*

**Supplementary Information**

Video Legend

**Supplementary Video 1.** Video clip representing an example of manipulative action (MAN_OBS) presented to participants during fMRI, showing an actor manipulating a small ball.

**Supplementary Video 2.** Video clip representing an example of movement (MOV_OBS) presented to participants during fMRI, showing an actor performing simple finger tapping movements.

Supplementary Methods

Imaging Parameters

Anatomical T1-weighted and functional T2*-weighted MR images were acquired with a 3 T General Electric scanner (MR750 Discovery) equipped with an 8-channel receiver head-coil. A high-resolution 3D isotropic T1-weighted-images sequence (called BRAVO, BRA in Volume) was acquired as anatomical reference. Its acquisition parameters were as follows: 196 slices, 280×280 matrix with a spatial resolution of 1×1×1 mm, TR = 9700 ms, TE = 4 ms, FOV = 252 x 252 mm; flip angle = 9°. Functional volumes were acquired either while participants performed the action observation task and the action execution task with the following parameters: forty axial slices of functional images covering the whole-brain acquired using a gradient-echo echo-planar imaging (EPI) pulse sequence, slice thickness = 3 plus interslice gap = 0.5 mm, 64×64×37 matrix with a spatial resolution of 3.5×3.5×3.5 mm, TR = 3000 ms, TE = 30 ms, FOV = 205 x 205 mm^2^, flip angle = 90°, in plane resolution = 3.2 x 3.2 mm^2^.

fMRI data preprocessing and analysis

Data processing was performed with SPM12 (Wellcome Department of Imaging Neuroscience, University College, London, UK; <http://www.fil.ion.ucl.ac.uk/spm>) running on MATLAB R2018a (The Mathworks, Inc.). Structural images were manually centred and reoriented with functional images to the anterior-posterior commissure axis. The first four EPI volumes of each functional run were discarded to allow the magnetization to reach a steady state. For each subject, all volumes were slice timing corrected, spatially realigned to the first volume of the first functional run and un-warped to correct for between-scan motion. Motion parameters were used as predictors of no interest in the model to account for translation and rotation along the three possible dimensions as determined during the realignment procedure. Individual dataset was excluded if excessive head motion was observed (translation > 3 mm or rotation > 3°). T_1_-weighted image was segmented into grey, white and cerebrospinal fluid and spatially normalized to the Montreal Neurological Institute (MNI) space. Spatial transformation derived from this segmentation was then applied to the realigned EPIs for normalization and re-sampled in 2×2×2 mm^3^ voxels using trilinear interpolation in space. All functional volumes were then spatially smoothed with a 6-mm full-width half-maximum isotropic Gaussian kernel (FWHM).

The statistical significance of the estimated evoked hemodynamic response (HRF) was assessed using *t*-statistic in the context of a General Linear Model (GLM). At first-level, the model combined the two action observation runs, modelling three predictors corresponding to experimental condition, control condition and response to catch-trials (MAN_OBS, MOV_OBS, and Response), six predictors obtained from the motion correction in the realignment process to account for voxel intensity variations caused by head-movement, and one constant regressor per run. All predictors, except for *Response*, included the 5 consecutive videos, which were modelled as one single epoch lasting 15 s. Catch-trials were modelled as consecutive blocks, lasting 15 s each, including the effective response time (3 s) and a signal-denoising period (12 s) to separate the motor component from subsequent processing. Contrasts derived from parameter estimates were calculated and entered into a flexible factorial within-subjects analysis of variance (ANOVA). Specific effects were tested using *t* statistical parametric maps (SPMt), with degrees of freedom corrected for non-sphericity at each voxel^1^. Data corresponding to action execution task were analysed using a different GLM model with two predictors (MAN_EXE, MOV_EXE), corresponding to 15 s execution blocks, convolved with the HRF.

1. Friston, K. J. *et al.* Classical and Bayesian inference in neuroimaging: Applications. *Neuroimage* **16**, 484–512 (2002).
